# Facile Synthesis and Characterization of CeO_2_-Nanoparticle-Loaded Carboxymethyl Cellulose as Efficient Protective Films for Mild Steel: A Comparative Study of Experiential and Computational Findings

**DOI:** 10.3390/polym14153078

**Published:** 2022-07-29

**Authors:** Mohamed Gouda, Mai M. Khalaf, Manal A. A. Al-Shuaibi, Ibrahim M. A. Mohamed, Kamal Shalabi, Reda M. El-Shishtawy, Hany M. Abd El-Lateef

**Affiliations:** 1 Department of Chemistry, College of Science, King Faisal University, Al-Ahsa 31982, Saudi Arabia; mmkali@kfu.edu.sa (M.M.K.); 218038147@kfu.edu.sa (M.A.A.A.-S.); 2 Chemistry Department, Faculty of Science, Sohag University, Sohag 82524, Egypt; 3 Department of Chemistry, College of Science and Humanities in Al-Kharj, Prince Sattam bin Abdulaziz University, Al-Kharj 11942, Saudi Arabia; dr-kamal@mans.edu.eg; 4 Chemistry Department, Faculty of Science, Mansoura University, Mansoura 35516, Egypt; 5 Chemistry Department, Faculty of Science, King Abdulaziz University, Jeddah 21413, Saudi Arabia; 6 Dyeing, Printing and Textile Auxiliaries Department, Institute of Textile Research and Technology, National Research Centre, 33 EL Buhouth St., Dokki, Giza 12622, Egypt

**Keywords:** nanocomposite, CMC, ceria NPs, coated films, theoretical modeling, material characterization

## Abstract

Corrosion is considered to be the most severe problem facing alloys and metals, one that causes potentially dangerous industrial issues such as the deterioration of buildings and machinery, and corrosion in factory tanks and pipelines in petroleum refineries, leading to limited lifetime and weak efficacy of such systems. In this work, novel CeO_2_-nanoparticle-loaded carboxymethyl cellulose (CMC) was successfully prepared by using a simple method. The structural configuration of the prepared CeO_2_-nanoparticle-loaded CMC was investigated by FE-SEM/EDX, TEM, FT-IR, and thermal analyses. The corrosion protection proficiency of uncoated and coated mild steel with CeO_2_-CMC systems in 1.0 M HCl solutions was studied by E_OCP_-time, EIS, and PDP tools. Moreover, the relationship between the structure of coating films and their corrosion protection was confirmed by DFT calculation and MC simulation. The obtained findings from the studied methods showed that the prepared CeO_2_-CMC-coated films reported high corrosion resistance. The protection capacity augmented with ceria presents an increase of up to 3% to achieve 98.4%. DFT calculation and MC simulation confirmed the influence of the chemical construction of coated films on its protection capacity, which was in accordance with the experimental results.

## 1. Introduction

Nanocomposite materials have emerged as a novel and superior alternative for numerous research and production objectives in recent years. Nanocomposites comprise heterogeneous films with chemically distinct phases or one phase isolated or embedded in another. There are either two separate crystalline phases, one crystalline and the other amorphous, or two distinct crystalline phases. In general, nanocomposite coatings consist of one host particle and another embedded homogeneously in it, one or both of which are nanoscale [1]. 

Nanocomposite coatings have unique mechanical, electronic, magnetic, and optical properties that make them useful and striking for a variety of engineering applications, including high-speed machining, tooling, optical devices, magnetic storage tools, and automation, aerospace, and biomedical applications. This is all due to their unique mechanical, electronic, magnetic, and optical properties arising from their small size. As a result, they are a rapidly growing area of interest that uses a variety of nanotechnologies and characteristics. In order that models and their various interfaces may be created, films can be synthesized with better outcomes and strength for less money. Furthermore, there is room in these coatings to achieve innovative and different functionalities, which can lead to pioneering properties [2,3,4,5].

Corrosion is one of the most pressing issues facing educational and industrial sectors worldwide since it has a detrimental impact on the economies of both emerging and established countries [6,7]. Corrosion caused by carbon steel in an acidic environment is mitigated by applying corrosion inhibitors in small amounts [8,9,10,11,12]. Organic compounds containing hetero-atoms, such as sulfur [13], oxygen [14], nitrogen [15], and phosphorous, comprise the bulk of commercially available acid inhibitors. Many heterocyclic compounds with nitrogen atoms have been discovered to be effective steel corrosion inhibitors in acidic settings [16,17,18].

With the prevalent use of “green chemistry” in all kinds of technology, science, and engineering, common morals aimed at diminishing pollution and increasing the usage of environmentally friendly chemicals has affected corrosion science, especially in terms of the use of inhibitors that originate from natural resources and biomasses [19]. Plants as natural products have lately been advocated [20] as green corrosion inhibitors due to their low cost, environmental acceptability, abundance, and effectiveness as anticorrosion agents. Bioactive substances such as tannins, phenols, flavonoids, acids, alkaloids, catechins, terpenoids, amino acids and proteins, polysaccharides, and vitamins are abundant [20].

Because of the value of metals and alloys, it is critical to investigate the causes of corrosion and how to prevent it. Corrosion behavior in metals and alloys is commonly studied using HCl and H_2_SO_4_ corrosive media. These acids are widely employed during acid pickling, chemical cleaning, and rust cleaning, resulting in metal loss [21]. From production to usage, most corrosion-resistant organic chemicals are costly, dangerous, and poisonous, which is why ongoing research is undertaken to limit their use to a large extent [22]. Natural corrosion inhibitors, which are biodegradable, affordable, and environmentally acceptable, are an economical and easy solution in this area. Various scientists and engineers employed components produced from the plant, such as root, fruit, flower, stem, gum, and water, as green corrosion inhibitors in the past [23]. Polysaccharides are natural polymers that are employed as metal corrosion inhibitors because they are ecologically benign, biodegradable, affordable, and stable. These are low-cost, renewable, and widely accessible, and they include OH groups, heteroatoms, and alkyl chain lengths, making them easy to deposit on metal surfaces [24,25,26,27,28].

Polymers derived from natural sources have the advantage of being renewable and decomposable [29], for example, natural polymers such as carboxymethyl cellulose (CMC) [30], starch [31], xanthan gum [32], chitosan [33], gum [34], iota-carrageenan [35], pectin, and gellan gum [36]. The primary obstacle is the inability of certain polymers to dissolve readily in an aqueous media, as well as their proclivity for breaking down at high temperatures [37]. As a result, some of them have limited or no protective capabilities. For example, Umoren et al. found that 0.5 g of carboxymethyl cellulose in an H_2_SO_4_ solution could only provide 64.8 percent protection on a mild steel surface [29]. Moreover, a novel carboxymethyl cellulose containing copper, iron, and nickel nanoparticles was prepared via an in situ deposition approach. This systematic approach was used to prepare a new sustainable and eco-friendly inhibitor nanocomposite applied in the field of anti-corrosion applications [38].

Superhydrophobic surfaces easily repel liquids and have attracted global interest from researchers, laypeople, and businesses [39]. To address this issue, Gouda et al. developed a superhydrophobic film based on carboxymethyl cellulose, called Nonanyl carboxy methylcellulose grafted polyacrylamide (NCMC-g-PAAm-SB), which was used as a protective layer for AISI-stainless steel corrosion [40]. Plants, such as cabbage and lotus leaves, have shown that cellulose is a superb building component for superhydrophobic surfaces [41]. Cellulose is also appealing for industrial applications due to its low-cost, long-lasting material, with multilateral nanoparticles that allow for easy changes [42].

The present work aimed to prepare novel CeO_2_-nanoparticle-loaded carboxymethyl cellulose (CMC), and its corrosion resistance as coating films for mild steel in acidic chloride medium utilizing electrochemical methods including *E_cor_*-time, polarization plots (PD), and electrochemical impedance spectroscopy (EIS) was evaluated. The chemical structure and physical properties of the CeO_2_–CMC composite were also examined by field emission scanning electron microscopy (FESEM), transmission electron microscope (TEM), and thermal gravimetric analysis. Finally, DFT calculation and MC simulation were employed to elucidate the protection mechanism.

## 2. Materials and Methods

### 2.1. Preparation of CeO_2_-Nanoparticle-Loaded Carboxymethyl Cellulose (CeO_2_-CMC)

CeO_2_-CMC films were created by adding CeO_2_ nanoparticles (CeO_2_NPs) to aqueous CMC. In the absence of light, a three-necked flask was swirled for two hours with a mixed solution of CMC (1.5 g%) and CeO_2_ NPs (5 percent of CMC weight). In a Teflon petri dish, CeO_2_-CMC films with a thickness of 0.8 mm were cast over the course of 24 h at 60 °C. CeO_2_-CMC nanocomposite films were produced when the prepared film was desalted with deionized water and dried for 72 h at 50 °C.

### 2.2. Preparation of CeO_2_-CMC Coating Films

As substrate supplies, MS alloy samples with the following chemical formulations were applied: C (0.18%), Mn (0.08%), Cr (0.72%), Ni (0.01%), and Fe (remaining percent). These samples were gradually polished with emery papers No. 600–1800, degreased in ethanol, acetone, and bi-distilled H_2_O for 5.0 min each, and then dried by air. Using a dip-coating method, the MS alloy surface coating was structured. The electrode substrate was submerged in the sol for 2 min while being pulled out at a speed of 10 mm per minute. The specimen was dried out in an oven at 80 °C for 15 min after air-dehydrating it. In order to increase the coating thickness, the process from immersion to dehydration was repeated twice. Different CeO_2_ % (1.0, 2.0, 3.0, 4.0, 5.0, and 6.0) were used to coat the samples.

### 2.3. Characterization Techniques

Field emission scanning electron microscopy (FESEM) complemented with EDAX (Jeol, Tokyo, Japan) with a voltage of around 20 kV was used to scan the morphological and chemical characteristics of CMC-CeO_2_. Additionally, a transmission electron microscope (TEM) and selected area electron analysis (SAED) in a Jeol-1230 electron microscope running at 200 KeV (JEOL, Peabody, MA 01960, USA) was applied to confirm the particle size of the prepared composite. After that, an FT-IR analysis was performed by using a spectrophotometer from BRUKER (Ettlingen 76275, Germany) which was applied to study function groups of the designed composite after and before CeO_2_ incorporation within 4000–400 cm^−1^. The thermal gravimetric analysis of the synthesized composite was performed by using TGA Instruments (New Castle, DE 19720, USA) for both CMC and CMC-CeO_2_ composite and the temperature range was changed from 25 °C to 700 °C under a flow of oxygen at a rate of 10 °C/min. The studied CMC-CeO_2_ composite has an organic component in addition to an oxide one, and these parts behave differently in terms of their thermal characteristics. Therefore, oxygen was applied as a flowing reagent to study this difference. 

### 2.4. Corrosion Protection Measurements

All electrochemical investigations were implemented in an electrochemical cell containing 3-electrode systems of Ag/AgCl/KCl_(sat)_ (silver/silver chloride), Pt-sheet, and the uncoated and coated MS specimens as reference, as counter electrode and working electrodes. EIS experiments were conducted to assess the corrosion protection of uncoated and coated specimens in 15% HCl solution at 50 °C in 100 kHz to 1.0 Hz as a frequency range. A Potentiostat/Galvanostat/ZRA (Gamry 600, Warminster, PA, USA) instrument was used for EIS investigation. The EIS experiments were performed at 10 points/decade (scan frequency rate) with an open circuit potential (*E*_OCP_) of 45 min, and the RMS signal was changed between 2.0 and 10.0 mV. PDP investigations were made to measure the corrosion protection proficiency of the uncoated and coated MS specimens. The PDP experiments were executed in the potential range of ±250 mV vs. *E*_OCP_ at a scan rate of 0.2 mV/s. All tests were completed at least in duplicated specimens.

### 2.5. Computational Details

The energy optimization of the CeO_2_, CMC, and CeO_2_-CMC molecules was achieved in aqueous environments using DFT calculations with basis set BOP with ab initio, GGA method, and DNP 4.4 accomplished in the Dmol^3^ module in Materials Studio V.7.0 program [43]. The outcomes achieved from DFT calculations involving the lowest unoccupied molecular orbital (LUMO), the highest occupied molecular orbital (HOMO), the gap energy (Δ*E*), electronegativity (*χ*), hardness (*η*), global softness (*σ*), and number of electrons transferred (Δ*N*), ∆*E_back-donation_*, and dipole moment (*µ*) were calculated as follows [44]:

(1) $$\chi =\frac{-E_{HOMO}-E_{LUMO}}{2}$$

(2) $$\eta =\frac{1}{\sigma }=\frac{E_{LUMO}-E_{HOMO}}{2}$$

(3) $$\Delta N=\frac{\phi -\chi_{inh}}{2(\eta_{Fe}-\eta_{inh})}$$

(4) $$\Delta E_{back-donation}=\frac{-\eta }{4}$$

where *φ* is the function work of Fe (110), *χ_inh_* is the inhibitor electronegativity, and *η_Fe_* and *η_inh_* are the hardness of Fe (0 eV) and inhibitor, respectively. 

The most suitable adsorption arrangements of the CeO_2_-CMC molecule on the Fe (110) surface were achieved from MC simulations by employing the adsorption locator module in the Materials Studio V.7.0 program [45]. Primarily, the energy optimization of the adsorbate molecules was implemented via the COMPASS module [46]. Afterward, the adsorption of the CeO_2_-CMC, hydronium ions, Cl^−^ ions, hydronium ions, and water molecules onto the surface of Fe(110) was executed in a simulation box (37.24 Å × 37.24 Å × 59.81 Å) [47]. 

## 3. Results and Discussion

### 3.1. Material Characterizations

#### 3.1.1. SEM and TEM Analyses

**Figure 1:**
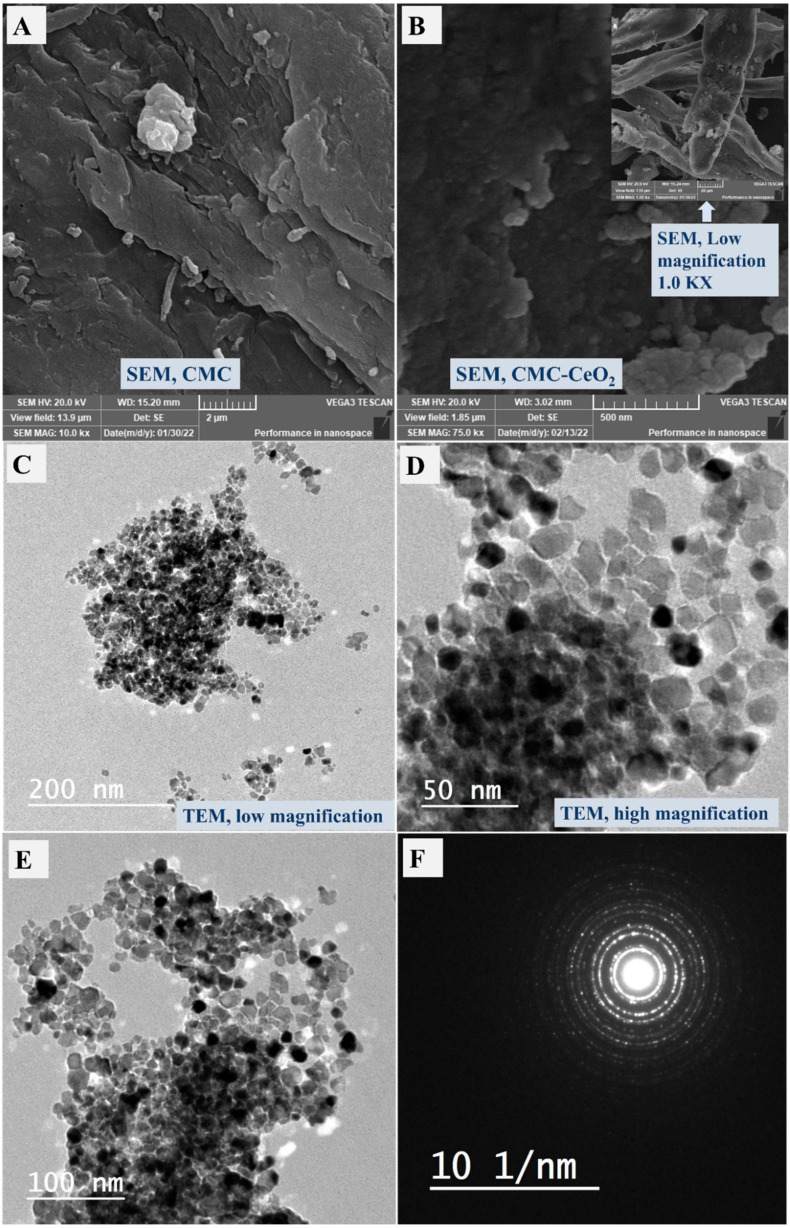
SEM of CMC (**A**) and CMC-CeO_2_ (**B**) containing low-magnification image in the inset of the figure, and TEM images of CMC-CeO_2_ composite at low (**C**) and high (**D**,**E**) magnification, and SAED image of CMC-CeO_2_ (**F**).

The SEM images of CMC and the synthesized CMC-CeO_2_ were studied at 75 KX as displayed in Figure 1A,B, respectively, in addition to TEM analysis, as shown in Figure 1C,D, at low and high magnification, respectively. For SEM analysis, the CMC image (Figure 1A) has a homogeneous character without clear isolated particles. After incorporation of CeO_2_, the SEM surface image becomes heterogeneous, having different size particles which indicate the successful incorporation of CeO_2_ onto the surface of CMC to design a CMC-CeO_2_ composite. The observed particles in the SEM of CMC-CeO_2_ were checked by TEM analysis at low and high magnifications (Figure 1C,D, respectively). Nanoparticles could be seen in both TEM images, especially at high magnification. Another TEM image is shown in Figure 1E, and the network structure of the utilized polymer (CMC) can be seen, which confirms the morphology of CMC-CeO_2_. The SAED image shown in Figure 1F clarifies the amorphous/crystallinity of the prepared polymer/oxide material and the crystalline phase of the prepared polymer/oxide composite, which confirms the presence of crystalline oxide even after incorporating it into the amorphous organic (CMC) template. Thus, the TEM in addition to SEM analysis proved that the prepared polymer/oxide composite has a heterogeneous surface morphology, containing nanoparticles. 

**Figure 2:**
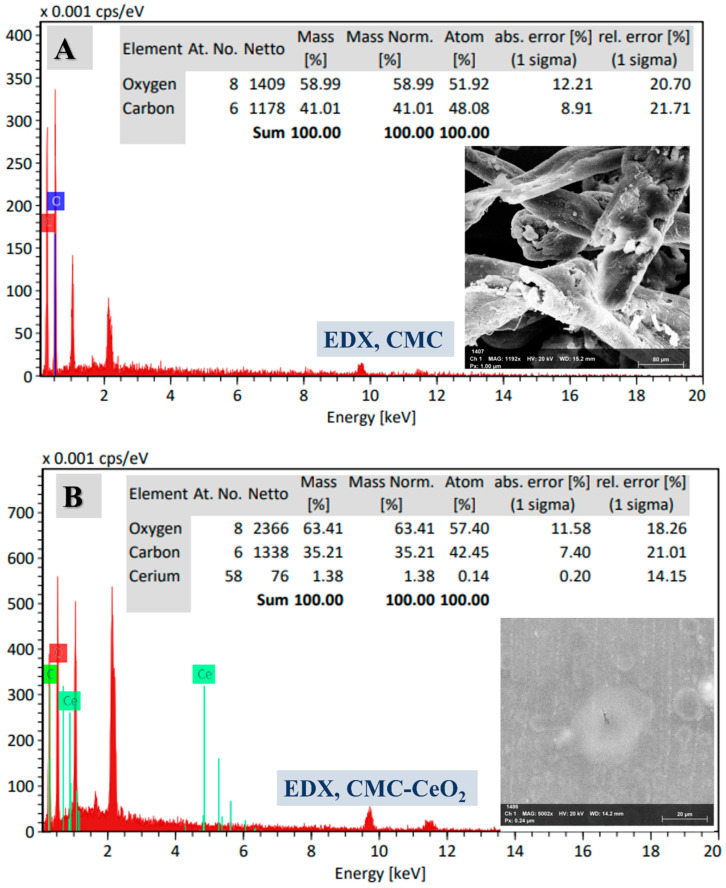
EDX of CMC (**A**) and CMC-CeO_2_ (**B**), and the inset is the chemical elemental analysis based on EDX peak analysis in addition to the EDX investigated area.

**Figure 3:**
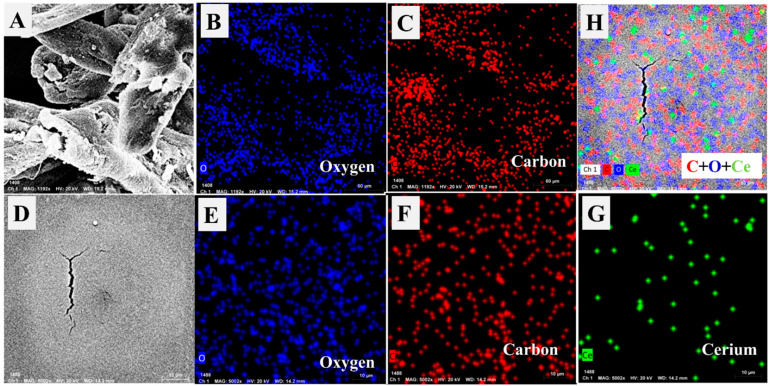
Chemical mapping by SEM equipment of CMC ((**A**): black photo; (**B**): oxygen map; (**C**): carbon map) and CMC-CeO_2_ ((**D**): black studied image; (**E**): oxygen map; (**F**): carbon map; (**G**): cerium map; (**H**): total survey map of all elements).

To investigate the chemical contents of the composite particles, EDX-SEM analysis was carried out for CMC and CMC-CeO_2_ as shown in Figure 2A,B, respectively. For CMC before the incorporation of CeO_2_ particles, the atomic percentages of O and C were detected at 58.99%, and 41.01%, respectively. After incorporation of the CeO_2_ particles, the atomic percentages of O, C, and Ce were observed at 63.41%, 35.21%, and 1.38%, respectively. The decrease in carbon content, as well as increased oxygen content, indicates that the chemical content changed to include more oxide from CeO_2_ incorporation. The mapping of C, O, and Ce was additionally studied for CMC and CMC-CeO_2_ as described in Figure 3A–G. The increase in O content and decrease in carbon content could be observed and confirmed in elemental mapping as explained in EDX-SEM analysis. Furthermore, the map of the Ce element was clear and distributed and checked in a total survey (Figure 3H). The total survey image indicates the successful incorporation of CeO_2_ by the homogeneous distribution of Ce in addition to O. The oxygen content is higher than that of cerium because oxygen is existent in CMC polymer besides ceria. The SEM, TEM, EDX, and elemental mapping confirm the preparation of CMC-CeO_2_ as a polymer/oxide composite. 

#### 3.1.2. FT-IR Analysis

**Figure 4:**
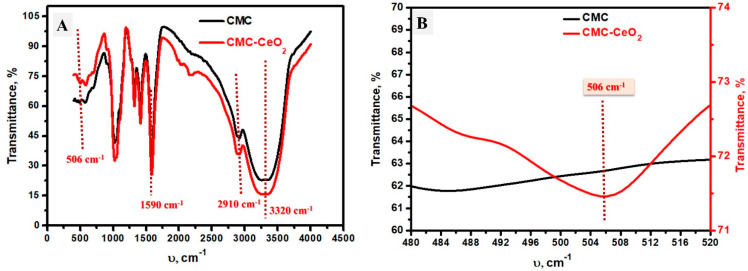
(**A**) FT-IR analysis of CMC and CMC-CeO_2_, (**B**) FT-IR in the frequency range of 480–520 cm^−1^.

The chemical bonds in CMC and the prepared CMC-CeO_2_ composite were investigated using FT-IR analysis as shown in Figure 4A. Both materials (CMC and CMC-CeO_2_ composite) mostly have the same chemical bonds which were indicated by the same locations of FT-IR peaks. The wide peak seen at 3320 cm^−1^ was ascribed to stretching vibration of OH stretching, while the peak at 2910 cm^−1^ referred to asymmetric CH_2_ stretching. The peaks at 1590 and 1419 could belong to the vibration of the C = O, and tensile vibrations of -C-O-C- bonds [48]. A small peak at 506 cm^−1^ was found in the CMC-CeO_2_ and not observed in CMC when using FT-IR, which could be due to the Ce-O vibration bond [49]; this is clarified in Figure 4B. In short, FT-IR spectroscopy confirms the chemical existence of CMC function groups in both CMC and CMC-CeO_2_ composite in addition to a small peak related to the Ce-O bond in the case of CMC-CeO_2_. 

#### 3.1.3. Thermal Properties

**Figure 5:**
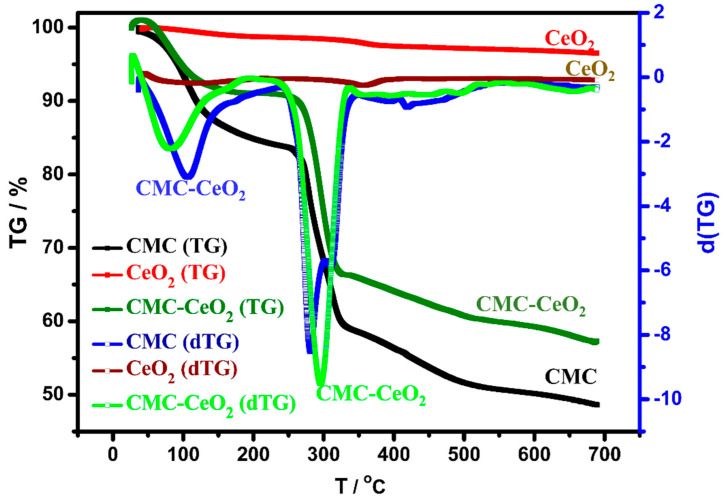
TGA analyses of CMC, CeO_2_, and CMC-CeO_2_ samples and their dTG analysis.

TGA and its differential (dTG) analyses were studied for the CMC-CeO_2_ composite and its components; CMC and CeO_2_ are described in Figure 5. For CeO_2_, the loss of weight is negligible. It is observed at 3.5% and could be due to the expected thermal stability of metal oxide (CeO_2_) up to 700 °C. In the case of the other investigated samples, CMC and CMC-CeO_2_, there are two clear exothermic peaks at 106 °C and 279 °C for CMC and a slight shift for CMC-CeO_2_. The exothermic peaks were seen at 99 °C and 297 °C in the case of CMC-CeO_2_. These exothermic peaks could be attributed to the evaporation of adsorbed water for the low-temperature peak (around 100 °C) and the decomposition of organic contents for the high-temperature peak (around 300 °C). The weight loss (%) of CMC, CeO_2_, and CeO_2_-CMC was found at 6.43%, 0.4%, and 5.33%, respectively, at 100 °C, which indicates the successful preparation of CeO_2_-CMC composite. Additionally, weight loss was found at 48.60% and 57.26% at 700 °C for CMC and CMC-CeO_2_, respectively. The difference between CMC and CMC-CeO_2_ in weight loss and exothermic peak positions indicates the interaction between the polymer content (CMC) with the oxide part (CeO_2_). This deduced interaction could provide higher thermal stability to the polymer contents, and so a shift to a higher temperature occurs.

### 3.2. OCP vs. Time and PDP Studies

**Figure 6:**
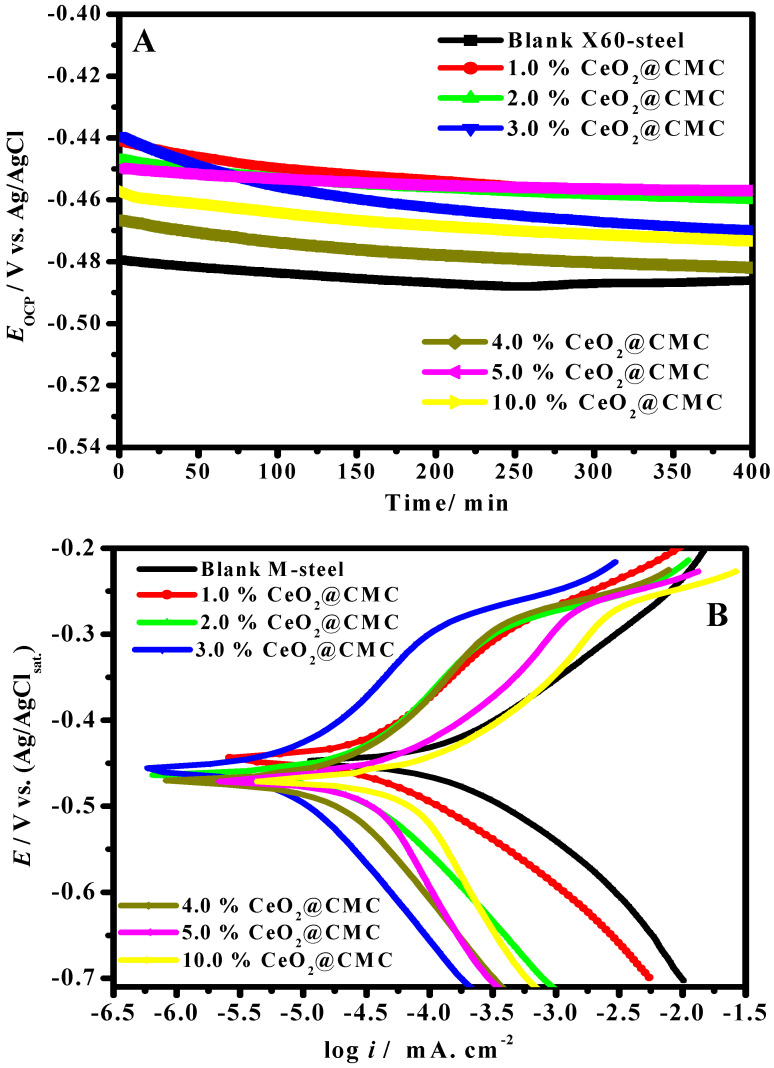
Change in OCP with time (**A**) and Tafel plots (**B**) in 1.0 M HCl solution for MS and coated with various values of %CeO_2_-CMC at 50 °C.

It is widely acknowledged that the OCP of a corrosion process is a property utilized as a thermodynamic constraint of electrochemical process inclination in a corrosive medium [50]. The corrosion potential vs. time could be changed due to the progress of the passive layer, resistance, or oxidation [51]. Figure 6A reveals the change in OCP with time in a molar HCl solution for pristine MS and coated samples with the various percentages of CeO_2_-CMC at 323 K. All coated samples at the preliminary time of immersion had further positive potential in comparison with the blank MS, which confirms that the coated MS samples are in a passive situation and, accordingly, well protected from the acidic solutions [52]. In general, the primary positive potential for coated CeO_2_-CMC layers is related to the reduction-oxidation route in the CMC matrix. These results showed that the coated CeO_2_-CMC films supported the development of a stable passive film, possibly in a process comparable to anodic protection. This confirms that the coated CeO_2_-CMC layers increase MS corrosion protection by increasing the nobility of metal.

The potentiodynamic polarization plots of the pristine MS and coated substrates with diverse ratio of % CeO_2_-CMC were completed after 50 min of immersion in molar hydrochloric acid at 323 K, as depicted in Figure 6B. It can be observed in Figure 6B that the anode and cathode branches were influenced as the % CeO_2_ increased in the coating layers, which revealed that these layers block the anodic corrosion of the MS and the hydrogen evolution of cathodic reaction [53,54,55]. Furthermore, the anodic and cathodic divisions shift to smaller current densities in the case of coated layers, which causes a decrease in the wear-active centers [56,57,58].

The coated and uncoated parameter values, such as corrosion potential (*E_cor_*), corrosion current density (*i_cor_*), and anodic and cathodic Tafel slopes (*β*_a_*, β*_c_), shown in Table 1 were ascertained by extrapolating Tafel slopes to the corrosion potential. The values of protection capability (*η*/%) were computed by determining the *i_cor_*, from the coated ($i_{cor}^{c}$) and uncoated ($i_{cor}^{0}$) by the following equation [59,60,61,62]:

(5) $$\mathrm{Protection}\;\mathrm{capability}\;/\%=\left(\frac{i_{cor}^{0}-i_{cor}^{c}}{i_{cor}^{0}}\right)\times 100$$

As shown in Table 1, the values of *E_cor_* in the presence of coatings films do not show obvious change, indicating that these films prevent both anodic corrosion and hydrogen evolution cathodic reactions, i.e., an increase in corrosion resistance. After the inclusion of ceria NPS in the CMC matrix, the MS surface shows a lower *i_cor_*, signifying a decline in the rate of corrosion of the CeO_2_-CMC coated samples. That is, the ceria NP-loaded carboxymethyl cellulose coatings provide enhanced corrosion resistance for the MS interface in a molar hydrochloric acid medium. Predominantly, 3.0%CeO_2_-CMC and 4.0%CeO_2_-CMC coating films display the lowest *i_cor_* among all the samples, indicating the extreme effectiveness of this layer, due to its uniform surface structure. The reserved protection properties can be ascribed to the thick surface structure and enriched adhesion after ceria are embedded in the CMC matrix, consequently decreasing the corrosive Cl^-^ ion permeation. The values of cathodic and anodic Tafel slopes (*β*_a_ and *β*_c_*)* in the case of coated samples were found to be greater than in the case of uncoated MS, suggesting that the ceria-CMC coating films change the hydrogen evolution reaction mechanism [63,64,65,66]. Furthermore, *β*_c_ values are also found to be lower than the analogous *β*_a_.

The findings presented in Table 1 show a remarkable decrease in values of *i_cor_* in the presence of CeO_2_ NPs in the CMC matrix compared with the coated surface with CMC alone, and this decrease is overcome by increasing the percentage of CeO_2_ NPs up to 3%, then there is an upsurge. The values of *i_cor_* decrease in the following order: uncoated MS (765.3 µA cm^−2^) > pristine CMC coating (278.6 µA cm^−2^) > 1.0%CeO_2_ coating (179.1 µA cm^−2^) > 2.0%CeO_2_ coating (79.6 µA cm^−2^) > 3.0%CeO_2_ coating (28.3 µA cm^−2^) < 4.0%CeO_2_ coating (44.4 µA cm^−2^) < 5.0%CeO_2_ coating (56.6 µA cm^−2^) < 10.0%CeO_2_ coating (107.9 µA cm^−2^). This suggests that ceria-CMC reduces the MS corrosion rate. The decline in *i_cor_* values in the presence of CeO_2_ embedded in the CMC matrix can be attributed to the impeding of the efficient centers present on the MS substrate [67]. The highest protection capacity of ~96.3% was documented in the case of the 3.0%CeO_2_-CMC-coated layer. The MS substrate coated with CeO_2_-CMC revealed reputable corrosion protection, which might be due to the well-intentioned dispersion of ceria in the CMC matrix. The use of a CeO_2_-embedded CMC matrix enhances the barrier features due to greater crosslinking of the CeO_2_-CMC composite. Ultimately, the existence of CeO_2_ NPs embedded in the CMC structure decreases the pores available for H_2_O absorption and eliminates conductive paths in the coating film, inventing its barrier characteristics.

### 3.3. EIS studies

**Figure 7:**
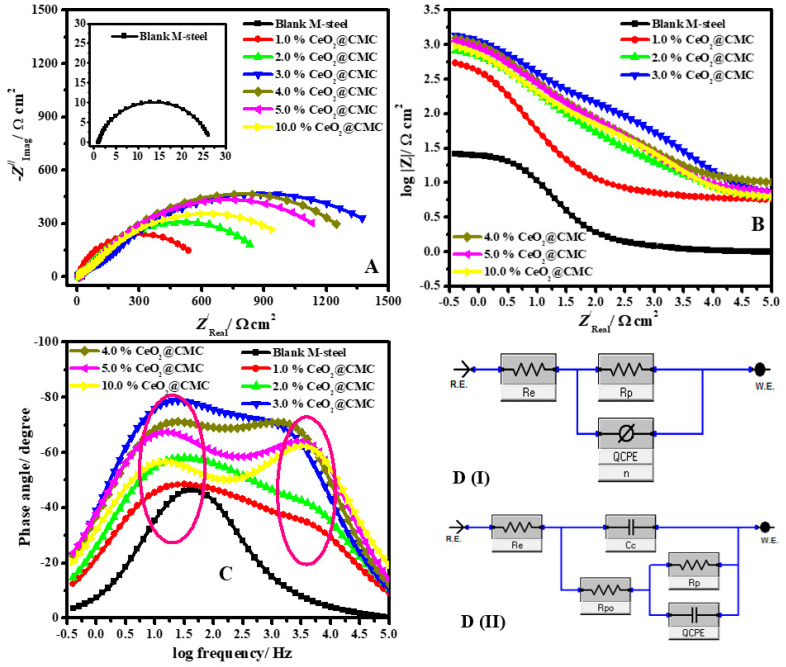
Nyquist (**A**), Bode (**B**), Bode phase (**C**) plots 1.0 M HCl solution for MS and coated with various values of %CeO_2_-CMC at 50 °C and (**D**) EEC for uncoated (DI) and coated (DII) systems.

To clarify the steel kinetic processes and the surface features of M-steel, the EIS investigation was conducted on uncoated and coated M-steel in hydrochloric acid solution. The Nyquist (A), Bode (B), and Bode phase (C) diagram 1.0 M HCl solution for MS and coated with various values of %CeO_2_-CMC at 50 °C are represented in Figure 7A–C respectively.

The Nyquist diagram (Figure 7A) in the case of pristine MS bare shows a single capacitive loop (one semicircle), which is ascribed to the corrosion process of charge–transfer. Nevertheless, in the case of coated MS surfaces with different values of %CeO_2_-CMC nanocomposites, two capacitive loops are discerned as shown in Figure 7A. At relatively high-frequency (HF) areas, the inductive-loop can be clarified as the coating film capacitance (*Q*_coat_, *CPE*_coat_) and the resistance of coating film (*R*_c_) [49]. However, at low frequency (LF), the capacitive-loop areas might be attributed to the polarization–resistance (*R*_p_) in parallel with the capacitance of the double layer (*Q*_dl_, *CPE*_dl_) [50]. The occurrence of double capacitive loops for coated surfaces has been recorded previously [51,52]. The capacitive loops are not precise semicircles and are depressed to some degree. This is associated with the frequency dispersion effect as a result of the roughness and inhomogeneity of the metal surface.

The diameter of Nyquist plots in the case of all coated layers with different ceria percentages was larger than that of the uncoated MS surface. This suggests that the %CeO_2_–CMC-coated films create a protective layer on the steel interface, consequently increasing the impedance of corrosion by the metal surface. This diameter of the semicircle increases with increasing the CeO_2_ percent in the coating structure until 3%CeO_2_ then declines to 10%CeO_2_ (Table 2), which indicates that the steel protection is directly dependent on ceria percent. The coated layers of %CeO_2_-CMC designed the protective adsorbed films on the metal surface by forming a robust bond among N atoms in the CeO_2_-CMC film and the metal substrate. These layers are designed on the metal interface and proficiently block it from the corrosive chloride ions [68]. The Bode impedance modulus (Figure 7B) shows linear portions at middle frequencies, and the linearity is further evident in the case of the coated films, implying greater slopes than the uncoated metal [69]. 

In order to fit the EIS data, the equivalent circuit (EEC) was used as depicted in Figure 7D (uncoated (DI) and coated (DII)). The model utilized to fitting the EIS findings contains constant phase-element (CPE); *R*_e_ (solution resistance); polarization resistance (*R*_p_) (*R*_p_ = *R*_L_ (layer resistance) + *R*_ct_ (charge transfer resistance)), i.e., a simple Randles EEC accompanied by *C*_c_ (coating pseudo-capacitance); and *R*_po_ (electrical pore resistance) in the presence of coated films. The calculated electrochemical parameters such as *R*_p_, *R*_po_, CPE, and *n* were fitted by using the Z-View program and are listed in Table 2. The protection proficiencies (%*η_e_*) were calculated from the following equation [70]:

(6) $$\eta_{e}/\%=\left(1-\frac{R_{p}^{0}}{R_{p}^{c}}\right)\times 100=\theta \times 100$$

where *θ* is the surface coverage part; $R_{p}^{0}$ and $R_{p}^{c}$ describe polarization resistances in the case of uncoated and coated substrates, respectively. To provide a more accurate fitting, CPE was calculated rather than the pure capacitance element; the double-layer is created by the film on the metal surface functioning as CPE [71]. The impedance of CPE is estimated from the following equation [71]:

(7) $$Z_{CPE}=Y_{0}^{-1}{\left(j\omega \right)}^{-n}$$

where *Y*_0_ represents the magnitude of CPE, *ω* is angular frequency, *j* is the $\sqrt{-1}$, and *n* represents the phase shift. The lower value of *n* (Table 2) for uncoated MS reflects the surface heterogeneity caused by the steel’s roughening surface due to corrosion. Nonetheless, in the presence of films coated with CeO_2_-CMC, the *n* values were increased, signifying declining heterogeneity of surface due to the structure of combined ceria and CMC-coated films. In addition, the *R*_po_ values were found to increase with the ceria content up to 3%, after which they decreased. The highest *R*_po_ value was attained for the sample coated with 3%CeO_2_-CMC film, confirming that this is the minimal porous coating film (Table 2). 

A considerable increase in the *R*_p_ was noticed in the presence of the coated films compared with the uncoated sample (Table 2). The *R*_p_ increases with increasing the ceria percent in the CMC matrix, resulting in enhanced coating protection. This phenomenon is due to the structure of coated films on the metal surface. The characteristic structures of ceria nanoparticles prevent the corrosive ions from infiltrating the metal surface and increase the resistance of coating corrosion, consequently impeding more charge and mass transfer. As shown in Table 2, the *η_e_*/% and *R*_p_ order of diverse coating layers throughout the entire inspection method is as follows: 3.0%CeO_2_-CMC (98.4%) > 4.0%CeO_2_-CMC (98.1%) > 5.0%CeO_2_-CMC (98.0%) > 10.0%CeO_2_-CMC (97.7%) > 2.0%CeO_2_-CMC (97.2%) > 1.0%CeO_2_-CMC (95.8%).

**Figure 8:**
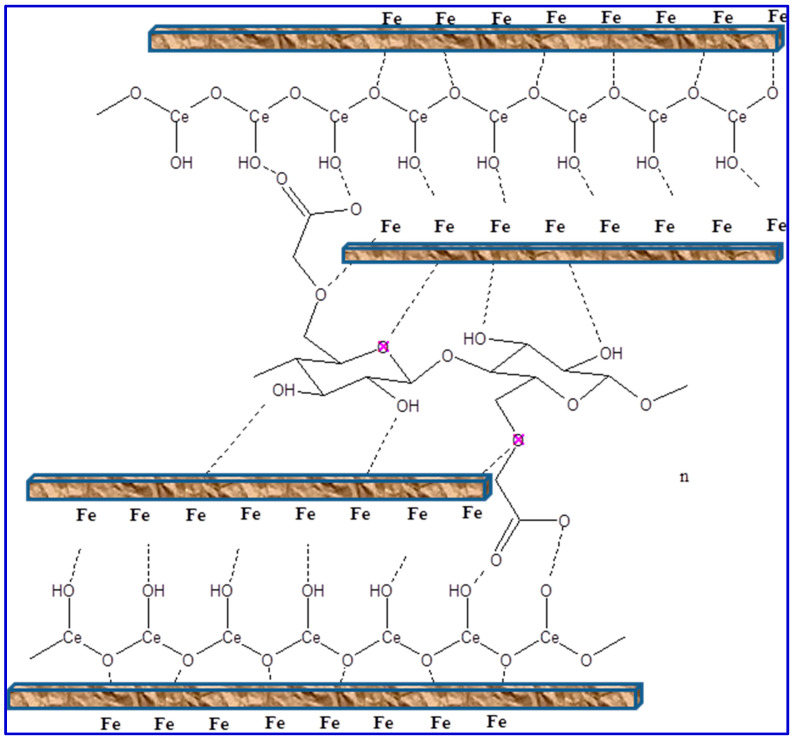
Schematic depiction of the CeO_2_-CMC molecule adsorption onto the MS surface in HCl solution.

The values of CPE for the blank and coated samples are presented in Table 2. The CPE values for coated films decrease in comparison with the uncoated surface because of the protection efficacy improvement provided by the CeO_2_-CMC films. The greater value of *R*_p_ with a smaller CPE value shows the superior corrosion impedance of the prepared coating layers [72]. It could be detected that the CPE values dropped from 123.61 µΩ^−1^ s^n^ cm^−2^ to 45.24, 34.65, 21.37, 24.34, 26.89, and 31.23 µΩ^−1^ s^n^ cm^−2^ in the presence of coated films with 1.0%CeO_2_-CMC, 2.0%CeO_2_-CMC, 3.0%CeO_2_-CMC, 4.0%CeO_2_-CMC, 5.0%CeO_2_-CMC, and 10.0%CeO_2_-CMC. This decrease in CPE values is ascribed to the augmentation in the thickness of the electrical double-layer and/or the decrease in local-dielectric constant, indicating that ceria-CMC composite decreases the corrosion rate by steady adsorption at the metal/solution interface [73]. The improvement in *R*_p_ is consistent with the increase in *R*_po_ and decrease in CPE values of the coating films, confirming the anticorrosion capability of the prepared films. The steadiness of the coated layers is due to the robust adsorption of CMC active centers on the metal surface through the contribution of lone electron pairs of oxygen atoms to the empty d-orbital of Fe ions to form a coated layer of CMC molecules that operates by ceria NPs on the electrode substrate, as shown in Figure 8.

### 3.4. Computational Calculations (DFT)

**Figure 9:**
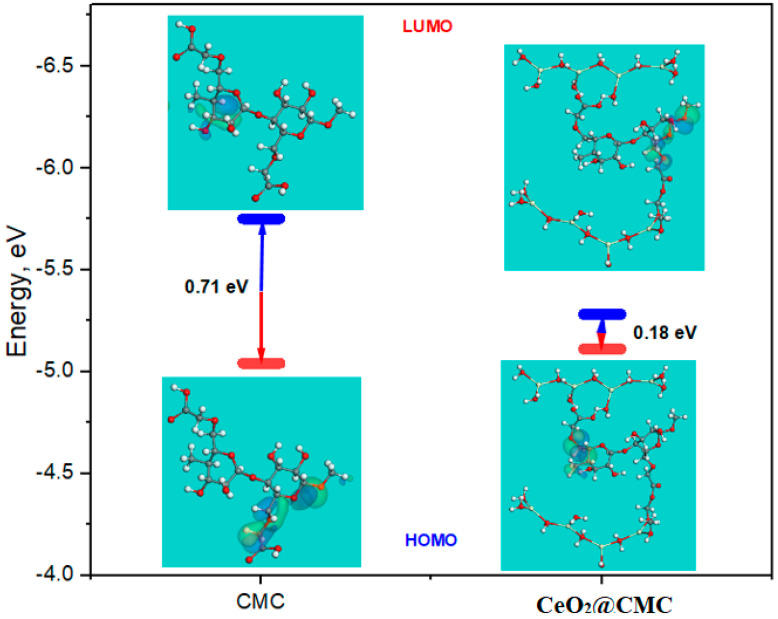
Energy diagram of the Frontier molecular orbitals for the CMC and CeO_2_-CMC molecules utilizing DFT method.

Figure 9 presents an energy diagram of the Frontier molecular orbitals (FMO) for CMC and CeO_2_-CMC molecules, and the associated theoretical parameters are listed in Table 3. It is evident that the HOMO level for CeO_2_-CMC molecule was situated on the nitrogen and oxygen atoms, as shown in Figure 9, which are desired sites for electrophilic attacks on the MS surface. Furthermore, the Δ*E* (energy gap) is a decisive parameter to boost the corrosion protection capability of the inhibitor molecule, i.e., it enhances as the Δ*E* value is reduced [74]. As shown in Table 3, the CeO_2_-CMC molecule has a smaller Δ*E* value (0.18 eV) than CeO_2_ and CMC molecules, which develops the great tendency of CeO_2_-CMC molecules to be adsorbed onto the steel interface. 

Usually, corrosion inhibitors have low electronegativity (*χ*), indicating the inhibitors’ aptitude for electron provision to the metal surface [70]. However, high electronegativity (*χ*) indicates an inhibitor’s ability to receive the electron from steel interface atoms (i.e., back-donation) and create a powerful bond with the MS surface [75]. The electronegativity of CeO_2_, CMC, and CeO_2_-CMC molecules are moderately high (about 5) as shown in Table 1, revealing the back-donation propensity of the CeO_2_, CMC, and CeO_2_-CMC molecules to create a durable bond with the MS surface. 

Additionally, softness (*σ*) and hardness (*η*) are important indicators to assess the stability and reactivity of the inhibitor molecule, i.e., soft molecules have protection proficiency greater than that of hard molecules through the effortless transfer of electrons to the steel interface, forming an adsorbed layer that can be considered as an effective corrosion inhibitor [71]. As shown in Table 3, the CeO_2_-CMC molecule has a larger *σ* value (11.24) and lower *η* value (0.09) than CMC molecules, while the CeO_2_ molecule has a low *σ* value (0.30) and high *η* value (3.39), indicating the low electron contribution capability of CeO_2_ molecules in contrast with CeO_2_-CMC molecules, which have an efficient contribution of electrons to the steel surface and excellent protection capability.

Furthermore, the electron-donating or accepting capacity of the inhibitor molecule can be clarified by the fraction of electron transfer and Δ*E_back__-donation_*. As follows, if the Δ*N* values are more than 0, the electron transfer is probable from inhibitor to MS interface atoms, whereas, if the Δ*N* values are less than 0 zero, the electron transfer is possible from metal atoms to inhibitor molecule (i.e., back-donation) [72]. As shown in Table 3, the Δ*N* value for the CeO_2_@-CMC molecule is above zero (10.14) and higher than CeO_2_ and CMC molecules, revealing that the CeO_2_-CMC molecule is proficient in providing electrons to the MS surface. In addition, the Δ*E_back__-donation_* will be < 0 when *η* > 0, the electron will be transmitted to a molecule, followed by a back-donation from the molecule, and this is energetically desired [76]. In Table 3, the value of Δ*E_back__-donation_* for CeO_2_, CMC, and CeO_2_-CMC molecules are negative, which reveals that back-donation is favored for CeO_2_, CMC, and CeO_2_-CMC molecules and constructs a sturdy bond [77].

Similarly, the dipole moment is an imperative index that designates the corrosion protection capability of the inhibitor molecule [73]. Thus, the upsurge in dipole moment provides enhanced distortion energy, and augmentation in the adsorption of the inhibitor molecule on the MS surface implies a reinforcement in corrosion protection proficiency [78]. As shown in Table 3, the CeO_2_-CMC molecule has a greater dipole moment value (18.56 debye) than CeO_2_ and CMC molecules, which strengthens the additional proclivity for the CeO_2_-CMC molecule to be adsorbed onto the MS interface and improves the protection. Lastly, the protection proficiency of the CeO_2_-CMC molecule for corrosion of the MS surface is correlated with its molecular surface area. The corrosion protection capability improved as the molecular area was enhanced because of the increase in MS surface area shielded with adsorbed inhibitor molecules [79]. As shown in Table 3, the CeO_2_-CMC molecule had a greater molecular surface area (2741.52 Å^2^) than CeO_2_ and CMC molecules, which boosts the corrosion protection proficiency of the CeO_2_-CMC molecule. The theoretical outcomes are in accordance with the experimental findings.

### 3.5. MC Simulations

**Figure 10:**
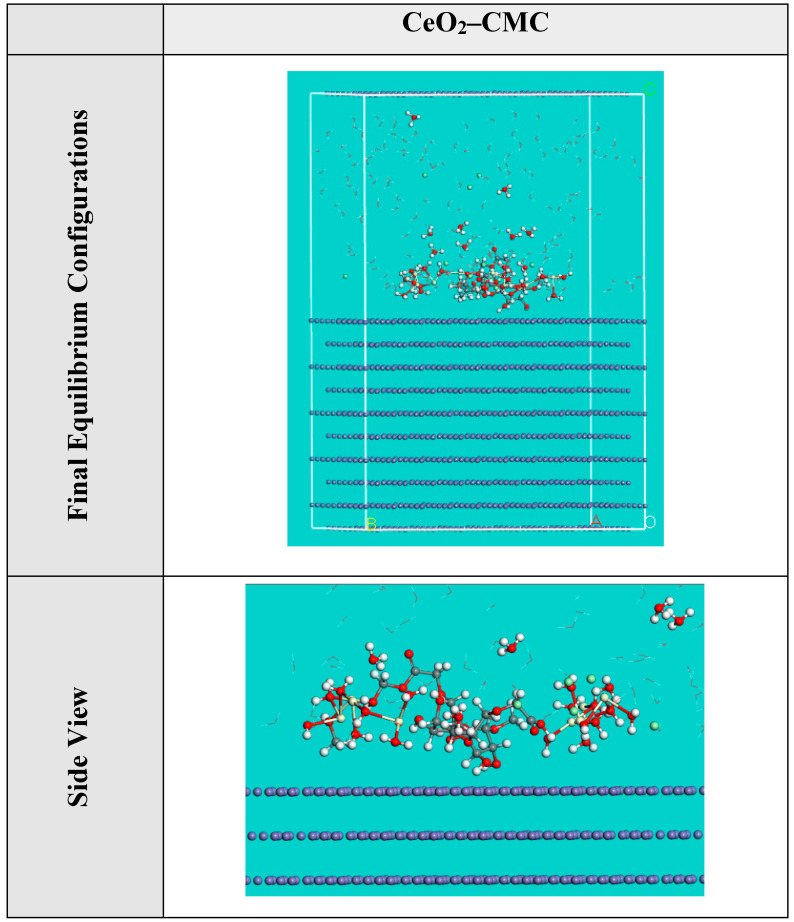
The highest proper adsorption arrangement for the CeO_2_-CMC molecule on Fe(110) accomplished by adsorption locator module.

MC simulations were designed to discern the adsorption of the inhibitor molecule onto the MS surface, and provide information about the protection mechanism. Thus, Figure 10 shows the most suitable adsorption conformations for the CeO_2_-CMC molecule on the MS interface in acidic medium which is displayed in a nearly flat arrangement, suggesting an improvement in the adsorption and highest surface coverage [80]. The evaluated results for the adsorption energies from the MC simulations are listed in Table 4. As seen in Table 4, the CeO_2_-CMC molecule has a high negative adsorption energy value of −2868.29 kcal mol^−1^, which indicates energetic adsorption of CeO_2_-CMC molecule onto the MS interface, generating a durable adsorbed film and shielding the MS surface from the corrosion. These outcomes are in accordance with the experimental results [81]. From Table 4, it is obvious that the adsorption energy value for CeO_2_-CMC molecule for the pre-geometry optimization step, i.e., unrelaxed (−2168.86 kcal mol^−1^) is highly negative and for the post-geometry optimization step, i.e., relaxed (−699.42 kcal mol^−1^) it is also negative, confirming a high protection proficiency for the CeO_2_-CMC molecule. 

The d*E*_ads_/d*N*_i_ values reveal the metal/adsorbates adsorption energy if one of the adsorbed species has been excluded [45]. The d*E*_ads_/d*N*_i_ value for the CeO_2_-CMC molecule (−372.13 kcal mol^−1^) is more negative than the d*E*_ads_/d*N*_i_ values for hydronium ions, chloride ions, and water molecules, which are −31.07, −61.07, and −15.26 kcal mol^−1^, respectively. Therefore, a more forceful adsorption of the CeO_2_-CMC molecule than hydronium ions, chloride ions, and water molecules enhances the exchange of hydronium ions, chloride ions, and water molecules by the CeO_2_-CMC molecule. Thus, the CeO2-CMC molecules are forcefully adsorbed onto the MS surface and create an efficient adsorbed protective layer, which shields the MS surface from corrosion, as confirmed by both experimental and computational studies.

## 4. Conclusions

In this study, CeO_2_-nanoparticle-loaded carboxymethyl cellulose was prepared, and its structure was categorized through FT-IR, TEM, FE-SEM/EDX, and thermal analysis. The SEM, TEM, EDX, and elemental mapping confirm the formation of CMC-CeO_2_ as a polymer/oxide composite. This prepared nanocomposite showed higher thermal stability. For the first time, CeO_2_-CMC composite with different CeO_2_ ratios displayed superior protective performance as a coating film for MS in HCl solutions. The protection capabilities of the as-prepared CeO_2_-CMC composites by electrochemical approaches followed the order of 3.0%CeO_2_-CMC (98.4%) > 4.0%CeO_2_-CMC (98.1%) > 5.0%CeO_2_-CMC (98.0%) > 10.0%CeO_2_-CMC (97.7%) > 2.0%CeO_2_-CMC (97.2%) > 1.0%CeO_2_-CMC (95.8%). The findings of the DFT calculations and MC simulations showed that coated films were robustly adsorbed onto the metal substrate, and theoretical parameters involving *E_HOMO_*, *E_LUMO_*, and Δ*E* were in agreement with the empirical data, which help elucidate the mechanism of adsorption of the CeO_2_-CMC composite onto metal surfaces. Consequently, CeO_2_-CMC composite is clearly a suitable eco-friendly coating film for MS corrosion in the pickling process.

## Figures and Tables

**Table 1:** PDP parameters for uncoated and coated MS in 1.0 M HCl solution at 50 °C.

Sample Description	*E_cor_*/ V vs. (Ag/AgCl)	*i_cor_*/ µA cm^−2^ ±SD	*β*_a_/ V dec ^−1^	*−β*_c_/ V dec ^−1^	*η*_Pc_/%
Blank MS	−0.447	765.3 ± 54.1	0.135	0.318	-
Pure CMC	−0.453	278.6 ± 16.7	0.153	0.334	63.6
1.0%CeO_2_-CMC	−0.445	179.1 ± 15.4	0.156	0.355	76.6
2.0%CeO_2_-CMC	−0.461	79.6 ± 4.6	0.149	0.359	89.6
3.0%CeO_2_-CMC	−0.456	28.3 ± 2.1	0.152	0.330	96.3
4.0%CeO_2_-CMC	−0.469	44.4 ± 3.6	0.145	0.345	94.2
5.0%CeO_2_-CMC	−0.470	56.6 ± 4.3	0.155	0.361	92.6
10.0%CeO_2_-CMC	−0.465	107.9 ± 8.5	0.145	0.352	85.9

**Table 2:** EIS fitting parameters for blank and coated M-steel in 1.0 M HCl solution at 50 °C.

Sample Description	*R*_s_/ Ω cm^2^	*CPE*_coat_ (*Q*_coat_)		*R*_po_/ Ω cm^2^ ±SD	*R*_p_/ Ω cm^2^ ±SD	*CPE*_dl_ (*Q*_dl_)		*θ*	*η_e_*/%
		*Y*_0_/ µ Ω^−1^ s^n^ cm^−2^	*n*			*Y*_0_/ µΩ^−1^ s^n^ cm^−2^	*n*		
Blank M-steel	0.95	-	-	-	27.7 ± 1.7	123.61	0.746	-	-
1.0%CeO_2_-CMC	4.71	221.72	0.859	23.1 ± 2.1	663.8 ± 40.6	45.24	0.874	0.958	95.8
2.0%CeO_2_-CMC	5.10	137.62	0.877	62.2 ± 4.5	1014.4 ± 60.3	34.65	0.855	0.972	97.2
3.0%CeO_2_-CMC	1.35	65.16	0.868	88.2 ± 7.4	1765.1 ± 116.4	21.37	0.905	0.984	98.4
4.0%CeO_2_-CMC	1.92	70.62	0.839	81.3 ± 5.5	1459.9 ± 78.8	24.34	0.911	0.981	98.1
5.0%CeO_2_-CMC	2.43	78.54	0.865	74.5 ± 4.8	1385.6 ± 76.1	26.89	0.915	0.980	98.0
10.0%CeO_2_-CMC	2.88	108.5	0.883	68.7 ± 5.1	1227.5 ± 65.2	31.23	0.899	0.977	97.7

**Table 3:** DFT parameters for CeO_2_, CMC, and CeO_2_–CMC molecules.

Parameters	CeO_2_	CMC	CeO_2_–CMC
*E_HOMO_* (eV)	−8.48	−5.75	−5.28
*E_LUMO_* (eV)	−1.71	−5.04	−5.11
Δ*E* = *E_LUMO_* − *E_HOMO_* (eV)	6.77	0.71	0.18
Electronegativity (*χ*)	5.10	5.39	5.20
Global hardness (*η*)	3.39	0.35	0.09
Global softness (*σ*)	0.30	2.83	11.24
The number of electrons transferred (Δ*N*)	0.28	2.27	10.14
∆*E_back-donation_*	−0.85	−0.09	−0.02
Dipole moments (*µ*) Debye	2.99	5.88	18.56
Molecular surface area (Å^2^)	427.35	1368.24	2741.52

**Table 4:** Data and descriptors computed by the MC simulations for the adsorption of the CeO_2_-CMC molecule on Fe(110).

Corrosion Systems	Adsorption Energy/ kcal mol^−1^	Rigid Adsorption Energy/ kcal mol^−1^	Deformation Energy/ kcal mol^−1^	d*E*_ads_/dN_i_: Inhibitor kcal mol^−1^	d*E*_ads_/d*N*_i_: Hydronium kcal mol^−1^	d*E*_ads_/d*N*_i_: Cl^−^ ions kcal mol^−1^	d*E*_ads_/d*N*_i_: Water kcal mol^−1^
Fe(110)	−2868.29	−2168.86	−699.42	−372.13	−31.07	−61.03	−15.26
CeO_2_-CMC							
Water							
Hydronium							
Cl^−^ ions							

## Data Availability

The raw/processed data generated in this work are available upon request from the corresponding author.

## References

[1] Sharma K., Goyat M.S., Vishwakarma P. (2020). Synthesis of Polymer Nano-composite coatings as corrosion inhibitors: A quick review. IOP Conf. Ser. Mater. Sci. Eng..

[2] Valipour M.N., Birjandi F.C., Sargolzaei J. (2014). Super-non-wettable surfaces: A review. Colloids Surf. A Physicochem. Eng. Asp..

[3] Manasa S., Jyothirmayi A., Siva T., Sarada B.V., Ramakrishna M., Sathiyanarayanan S., Gobi K.V., Subasri R. (2017). Nanoclay-based self-healing, corrosion protection coatings on aluminum, A356.0 and AZ91 substrates. J. Coatings Technol. Res..

[4] Maity S., Chatterjee A. (2013). Preparation and characterization of electro-conductive rotor yarn by in situ chemical polymerization of pyrrole. Fibers Polym..

[5] Verma C., Haque J., Quraishi M., Ebenso E.E. (2019). Aqueous phase environmental friendly organic corrosion inhibitors derived from one step multicomponent reactions: A review. J. Mol. Liq..

[6] Verma C., Ebenso E.E., Quraishi M. (2017). Ionic liquids as green and sustainable corrosion inhibitors for metals and alloys: An overview. J. Mol. Liq..

[7] Ahamad I., Quraishi M. (2009). Bis (benzimidazol-2-yl) disulphide: An efficient water soluble inhibitor for corrosion of mild steel in acid media. Corros. Sci..

[8] Obot I., Obi-Egbedi N. (2010). Adsorption properties and inhibition of mild steel corrosion in sulphuric acid solution by ketoconazole: Experimental and theoretical investigation. Corros. Sci..

[9] Shukla S.K., Quraishi M. (2009). 4-Substituted anilinomethylpropionate: New and efficient corrosion inhibitors for mild steel in hydrochloric acid solution. Corros. Sci..

[10] Behpour M., Ghoreishi S., Soltani N., Salavati-Niasari M., Hamadanian M., Gandomi A. (2008). Electrochemical and theoretical investigation on the corrosion inhibition of mild steel by thiosalicylaldehyde derivatives in hydrochloric acid solution. Corros. Sci..

[11] Qiu L.-G., Wu Y., Wang Y.-M., Jiang X. (2008). Synergistic effect between cationic gemini surfactant and chloride ion for the corrosion inhibition of steel in sulphuric acid. Corros. Sci..

[12] Hosseini S., Azimi A. (2009). The inhibition of mild steel corrosion in acidic medium by 1-methyl-3-pyridin-2-yl-thiourea. Corros. Sci..

[13] Ali S., Saeed M., Rahman S. (2003). The isoxazolidines: A new class of corrosion inhibitors of mild steel in acidic medium. Corros. Sci..

[14] Li W., Zhao X., Liu F., Hou B. (2008). Investigation on inhibition behavior of S-triazole–triazole derivatives in acidic solution. Corros. Sci..

[15] Obot I., Obi-Egbedi N., Umoren S. (2009). The synergistic inhibitive effect and some quantum chemical parameters of 2,3-diaminonaphthalene and iodide ions on the hydrochloric acid corrosion of aluminium. Corros. Sci..

[16] Herrag L., Hammouti B., Elkadiri S., Aouniti A., Jama C., Vezin H., Bentiss F. (2010). Adsorption properties and inhibition of mild steel corrosion in hydrochloric solution by some newly synthesized diamine derivatives: Experimental and theoretical investigations. Corros. Sci..

[17] Raman R.S., Siew W. (2010). Role of nitrite addition in chloride stress corrosion cracking of a super duplex stainless steel. Corros. Sci..

[18] Marzorati S., Verotta L., Trasatti S.P. (2018). Green Corrosion Inhibitors from Natural Sources and Biomass Wastes. Molecules.

[19] Popoola L.T. (2019). Organic green corrosion inhibitors (OGCIs): A critical review. Corros. Rev..

[20] Zakeri A., Bahmani E., Aghdam A.S.R. (2022). Plant extracts as sustainable and green corrosion inhibitors for protection of ferrous metals in corrosive media: A mini review. Corros. Commun..

[21] Sekine I., Nakahata Y., Tanabe H. (1988). The corrosion inhibition of mild steel by ascorbic and folic acids. Corros. Sci..

[22] Wang X. (2019). Rose, Gardenia, and Solanum Violaceum Extracts as Inhibitors of Steel Corrosion. Int. J. Electrochem. Sci..

[23] Mohammed H., Sobri S.B. (2018). Corrosion inhibition studies of cashew nut (*Anacardium occidentale*) on carbon steel in 1.0 M hydrochloric acid environment. Mater. Lett..

[24] Ferreira E.S., Giacomelli F.C., Spinelli A. (2004). Evaluation of the inhibitor effect of l-ascorbic acid on the corrosion of mild steel. Mater. Chem. Phys..

[25] Raja P.B., Sethuraman M.G. (2008). Natural products as corrosion inhibitor for metals in corrosive media—A review. Mater. Lett..

[26] Behzadnasab M., Mirabedini S., Kabiri K., Jamali S. (2011). Corrosion performance of epoxy coatings containing silane treated ZrO_2_ nanoparticles on mild steel in 3.5% NaCl solution. Corros. Sci..

[27] Li X., Deng S., Fu H., Mu G. (2009). Inhibition effect of 6-benzylaminopurine on the corrosion of cold rolled steel in H_2_SO_4_ solution. Corros. Sci..

[28] Bello M., Ochoa N., Balsamo V., López-Carrasquero F., Coll S., Monsalve A., González G. (2010). Modified cassava starches as corrosion inhibitors of carbon steel: An electrochemical and morphological approach. Carbohydr. Polym..

[29] Solomon M., Umoren S., Udosoro I., Udoh A. (2010). Inhibitive and adsorption behaviour of carboxymethyl cellulose on mild steel corrosion in sulphuric acid solution. Corros. Sci..

[30] Khalaf M.M., Tantawy A.H., Soliman K.A., El-Lateef H.M.A. (2020). Cationic gemini-surfactants based on waste cooking oil as new ‘green’ inhibitors for N80-steel corrosion in sulphuric acid: A combined empirical and theoretical approaches. J. Mol. Struct..

[31] Lavanya M., Murthy V.R., Rao P. (2020). Erosion corrosion control of 6061 aluminum alloy in multi-phase jet impingement conditions with eco-friendly green inhibitor. Chin. J. Chem. Eng..

[32] Mobin M., Rizvi M. (2016). Inhibitory effect of xanthan gum and synergistic surfactant additives for mild steel corrosion in 1 M HCl. Carbohydr. Polym..

[33] Umoren S.A., Banera M.J., Alonso-Garcia T., Gervasi C.A., Mirífico M.V. (2013). Inhibition of mild steel corrosion in HCl solution using chitosan. Cellulose.

[34] Bentrah H., Rahali Y., Chala A. (2014). Gum Arabic as an eco-friendly inhibitor for API 5L X42 pipeline steel in HCl medium. Corros. Sci..

[35] Fares M.M., Maayta A., Al-Mustafa J.A. (2012). Corrosion inhibition of iota-carrageenan natural polymer on aluminum in presence of zwitterion mediator in HCl media. Corros. Sci..

[36] Rajeswari V., Kesavan D., Gopiraman M., Viswanathamurthi P. (2013). Physicochemical studies of glucose, gellan gum, and hydroxypropyl cellulose—Inhibition of cast iron corrosion. Carbohydr. Polym..

[37] Tantawy A.H., Soliman K.A., El-Lateef H.M.A. (2020). Novel synthesized cationic surfactants based on natural piper nigrum as sustainable-green inhibitors for steel pipeline corrosion in CO_2_-3.5%NaCl: DFT, Monte Carlo simulations and experimental approaches. J. Clean. Prod..

[38] El-Lateef H.M.A., Albokheet W.A., Gouda M. (2020). Carboxymethyl cellulose/metal (Fe, Cu and Ni) nanocomposites as non-precious inhibitors of C-steel corrosion in HCl solutions: Synthesis, characterization, electrochemical and surface morphology studies. Cellulose.

[39] Tian Y., Su B., Jiang L. (2014). Interfacial Material System Exhibiting Superwettability. Adv. Mater..

[40] El-Lateef H.M.A., Gouda M., Shalabi K., Al-Omair M.A., Khalaf M.M. (2022). Superhydrophobic films-based nonanyl carboxy methylcellulose grafted polyacrylamide for AISI-stainless steel corrosion protection: Empirical explorations and computational models. J. Mol. Liq..

[41] Koch K., Barthlott W. (2009). Superhydrophobic and superhydrophilic plant surfaces: An inspiration for biomimetic materials. Philos. Trans. R. Soc. London. Ser. A Math. Phys. Eng. Sci..

[42] Klemm D., Kramer F., Moritz S., Lindström T., Ankerfors M., Gray D., Dorris A. (2011). Nanocelluloses: A New Family of Nature-Based Materials. Angew. Chem. Int. Ed..

[43] El-Lateef H.M.A., Sayed A.R., Shalabi K. (2021). Synthesis and theoretical studies of novel conjugated polyazomethines and their application as efficient inhibitors for C1018 steel pickling corrosion behavior. Surf. Interfaces.

[44] El-Lateef H.M., Abdallah Z.A., Ahmed M.S. (2019). Solvent-free synthesis and corrosion inhibition performance of Ethyl 2-(1,2,3,6-tetrahydro-6-oxo-2-thioxopyrimidin-4-yl)ethanoate on carbon steel in pickling acids: Experimental, quantum chemical and Monte Carlo simulation studies. J. Mol. Liq..

[45] Dehghani A., Mostafatabar A.H., Bahlakeh G., Ramezanzadeh B. (2020). A detailed study on the synergistic corrosion inhibition impact of the Quercetin molecules and trivalent europium salt on mild steel; electrochemical/surface studies, DFT modeling, and MC/MD computer simulation. J. Mol. Liq..

[46] Feng Y., Chen S., Guo W., Zhang Y., Liu G. (2007). Inhibition of iron corrosion by 5,10,15,20-tetraphenylporphyrin and 5,10,15,20-tetra-(4-chlorophenyl)porphyrin adlayers in 0.5M H_2_SO_4_ solutions. J. Electroanal. Chem..

[47] El-Lateef H.M.A., El-Beltagi H.S., Mohamed M.E.M., Kandeel M., Bakir E., Toghan A., Shalabi K., Tantawy A.H., Khalaf M.M. (2022). Novel Natural Surfactant-Based Fatty Acids and Their Corrosion-Inhibitive Characteristics for Carbon Steel-Induced Sweet Corrosion: Detailed Practical and Computational Explorations. Front. Mater..

[48] Karkhani R., Javanbakht V. (2022). A polyurethane foam membrane filled with double cross-linked chitosan/carboxymethyl cellulose gel and decorated with ZSM-5 nano zeolite: Simultaneous dye removal. Int. J. Biol. Macromol..

[49] Culica M.E., Chibac-Scutaru A.L., Melinte V., Coseri S. (2020). Cellulose Acetate Incorporating Organically Functionalized CeO_2_ NPs: Efficient Materials for UV Filtering Applications. Materials.

[50] Wang J., Zeng H. (2021). Recent advances in electrochemical techniques for characterizing surface properties of minerals. Adv. Colloid Interface Sci..

[51] Hamadi L., Kareche A., Mansouri S., Benbouta S. (2020). Corrosion inhibition of Fe-19Cr stainless steel by glutamic acid in 1M HCl. Chem. Data Collect..

[52] Rammelt U., Duc L.M., Plieth W. (2005). Improvement of protection performance of polypyrrole by dopant anions. J. Appl. Electrochem..

[53] El-Lateef H.M.A. (2016). Synergistic effect of polyethylene glycols and rare earth Ce^4+^ on the corrosion inhibition of carbon steel in sulfuric acid solution: Electrochemical, computational, and surface morphology studies. Res. Chem. Intermed..

[54] El-Lateef H.M.A., Ismael M., Mohamed I. (2015). Novel Schiff base amino acid as corrosion inhibitors for carbon steel in CO_2_-saturated 3.5% NaCl solution: Experimental and computational study. Corrosion Rev..

[55] El-Lateef H.M.A. (2015). Experimental and Computational Investigation on the Corrosion Inhibition Characteristics of mild steel by some novel synthesized imines in Hydrochloric acid Solutions. Corros. Sci..

[56] El-Lateef H.M.A., Tantawy A.H. (2016). Synthesis and evaluation of novel series of Schiff base cationic surfactants as corrosion inhibitors for carbon steel in acidic/chloride media: Experimental and theoretical investigations. RSC Adv..

[57] El-Lateef H.M.A., Abo-Riya M.A., Tantawy A.H. (2016). Empirical and quantum chemical studies on the corrosion inhibition performance of some novel synthesized cationic gemini surfactants on carbon steel pipelines in acid pickling processes. Corros. Sci..

[58] El-Lateef H.M.A., Soliman K.A., Tantawy A.H. (2017). Novel synthesized Schiff Base-based cationic Gemini surfactants: Electrochemical investigation, theoretical modeling and applicability as biodegradable inhibitors for mild steel against acidic corrosion. J. Mol. Liq..

[59] Aly K.I., Moustafa A.H., Ahmed E.K., El-lateef H.M.A., Mohamed M.G., Mohamed S.M. (2018). New Polymer Syntheses Part 60: A Facile Synthetic Route to Polyamides Based on Thieno [2,3-b]thiophene and Their Corrosion Inhibition Behavior. Chin. J. Polym. Sci..

[60] Saleh M.M., Mahmoud M.G., El-Lateef H.M.A. (2019). Comparative study of synergistic inhibition of mild steel and pure iron by 1- hexadecylpyridinium chloride and bromide ions. Corros. Sci..

[61] Sayed A.R., El-lateef H.M.A., Mohamad A.D.M. (2018). Polyhydrazide Incorporated with Thiadiazole Moiety as Novel and Effective Corrosion Inhibitor for C-Steel in Pickling Solutions of HCl and H_2_SO_4_. Macromol. Res..

[62] El-Lateef H.M.A., Khalaf M.M. (2019). Novel dispersed Tl_2_O_3_-SiO_2_/polyaniline nanocomposites: In-sit polymerization, characterization and enforcement as a corrosion protective layer for carbon-steel in acidic chloride medium. Colloids Surf. A.

[63] El-Lateef H.M.A. (2020). Corrosion inhibition characteristics of a novel salycilidene isatin hydrazine sodium sulfonate on carbon steel in HCl and a synergistic nickel ions additive: A combined experimental and theoretical perspective. Appl. Surf. Sci..

[64] El-Lateef H.M.A., Alnajjar A.O. (2020). Enhanced the protection capacity of poly(o-toluidine) by synergism with zinc or lanthanum additives at C-steel/HCl interface: A combined DFT, molecular dynamic simulations and experimental methods. J. Mol. Liq..

[65] El-Lateef H.M.A., Khalaf M.M., El-Lateef H.M.A., Khalaf M.M. (2020). Fabrication and characterization of alumina-silica/poly(o-toluidine) nanocomposites as novel anticorrosive epoxy coatings films on carbon steel. Microchem. J..

[66] El-Lateef H.M.A., Sayed A.R., Shalabi K. (2022). Studying the effect of two isomer forms thiazole and thiadiazine on the inhibition of acidic chloride-induced steel corrosion: Empirical and Computer simulation explorations. J. Mol. Liq..

[67] Tantawy A.H., Soliman K.A., El-Lateef H.M.A. (2021). Experimental and computational approaches of sustainable quaternary bisammonium fluorosurfactants for corrosion inhibition as protective films at mild steel/H_2_SO_4_ interface. Colloids Surf. A Physicochem. Eng. Asp..

[68] Ramezanzadeh M., Ramezanzadeh B., Mahdavian M., Bahlakeh G. (2020). Development of metal-organic framework (MOF) decorated graphene oxide nanoplatforms for anti-corrosion epoxy coatings. Carbon.

[69] Cao K., Yu Z., Yin D., Chen L., Jiang Y., Zhu L. (2020). Fabrication of BTA-MOF-TEOS-GO nanocomposite to endow coating systems with active inhibition and durable anticorrosion performances. Prog. Org. Coat..

[70] Palaniappan N., Cole I.S., Kuznetsov A.E. (2020). Experimental and computational studies of graphene oxide covalently functionalized by octylamine: Electrochemical stability, hydrogen evolution, and corrosion inhibition of the AZ13 Mg alloy in 3.5% NaCl. RSC Adv..

[71] Yesudass S., Olasunkanmi L.O., Bahadur I., Kabanda M.M., Obot I., Ebenso E.E. (2016). Experimental and theoretical studies on some selected ionic liquids with different cations/anions as corrosion inhibitors for mild steel in acidic medium. J. Taiwan Inst. Chem. Eng..

[72] Debab H., Douadi T., Daoud D., Issaadi S., Chafaa S. (2018). Electrochemical and quantum chemical studies of adsorption and corrosion inhibition of two new schiff bases on carbon steel in hydrochloric acid media. Int. J. Electrochem. Sci..

[73] Goyal M., Vashist H., Kumar S., Bahadur I., Benhiba F., Zarrouk A. (2020). Acid corrosion inhibition of ferrous and non-ferrous metal by nature friendly Ethoxycarbonylmethyltriphenylphosphonium Bromide (ECMTPB): Experimental and MD simulation evaluation. J. Mol. Liq..

[74] El-Lateef H.M., Shaaban S., Shalabi K., Khalaf M.M. (2022). Novel organoselenium-based N-mealanilic acids as efficacious corrosion inhibitors for 6061 aluminum alloy in molar HCl: In-silico modeling, electrochemical, and surface morphology studies. J. Taiwan Inst. Chem. Eng..

[75] Lukovits I., Kálmán E., Zucchi F. (2001). Corrosion Inhibitors—Correlation between Electronic Structure and Efficiency. Corrosion.

[76] Gao G., Liang C. (2007). Electrochemical and DFT studies of β-amino-alcohols as corrosion inhibitors for brass. Electrochim. Acta.

[77] El-Lateef H.M.A., Shalabi K., Tantawy A.H. (2020). Corrosion inhibition of carbon steel in hydrochloric acid solution using newly synthesized urea-based cationic fluorosurfactants: Experimental and computational investigations. New J. Chem..

[78] Oyebamiji A.K., Adeleke B.B. (2018). Quantum chemical studies on inhibition activities of 2,3-dihydroxypropyl-sulfanyl derivative on carbon steel in acidic media. Int. J. Corros. Scale Inhib..

[79] Toghan A., Gouda M., Shalabi K., El-Lateef H. (2021). Preparation, Characterization, and Evaluation of Macrocrystalline and Nanocrystalline Cellulose as Potential Corrosion Inhibitors for SS316 Alloy during Acid Pickling Process: Experimental and Computational Methods. Polymers.

[80] Shalabi K., Helmy A., El-Askalany A., Shahba M. (2019). New pyridinium bromide mono-cationic surfactant as corrosion inhibitor for carbon steel during chemical cleaning: Experimental and theoretical studies. J. Mol. Liq..

[81] Özcan M., Dehri I., Erbil M. (2004). Organic sulphur-containing compounds as corrosion inhibitors for mild steel in acidic media: Correlation between inhibition efficiency and chemical structure. Appl. Surf. Sci..

